# National Characteristics of Emergency Care for Children with Neurologic Complex Chronic Conditions

**DOI:** 10.5811/westjem.17834

**Published:** 2024-02-09

**Authors:** Kaileen Jafari, Kristen Carlin, Derya Caglar, Eileen J. Klein, Tamara D. Simon

**Affiliations:** * University of Washington, Department of Pediatrics, Seattle, Washington; † Seattle Children’s Research Institute, Center for Clinical and Translational Research, Seattle, Washington; ‡ University of Southern California, Keck School of Medicine, Department of Pediatrics, Los Angeles, California

## Abstract

**Introduction:**

Most pediatric emergency care occurs in general emergency departments (GED), where less pediatric experience and lower pediatric emergency readiness may compromise care. Medically vulnerable pediatric patients, such as those with chronic, severe, neurologic conditions, are likely to be disproportionately affected by suboptimal care in GEDs; however, little is known about characteristics of their care in either the general or pediatric emergency setting. In this study our objective was to compare the frequency, characteristics, and outcomes of ED visits made by children with chronic neurologic diseases between general and pediatric EDs (PED).

**Methods:**

We conducted a retrospective analysis of the 2011–2014 Nationwide Emergency Department Sample (NEDS) for ED visits made by patients 0–21 years with neurologic complex chronic conditions (neuro CCC). We compared patient, hospital, and ED visits characteristics between GEDs and PEDs using descriptive statistics. We assessed outcomes of admission, transfer, critical procedure performance, and mortality using multivariable logistic regression.

**Results:**

There were 387,813 neuro CCC ED visits (0.3% of 0–21-year-old ED visits) in our sample. Care occurred predominantly in GEDs, and visits were associated with a high severity of illness (30.1% highest severity classification score). Compared to GED visits, PED neuro CCC visits were comprised of individuals who were younger, more likely to have comorbid conditions (32.9% vs 21%, *P* < 0.001), and technology assistance (65.4% vs. 45.9%) but underwent fewer procedures and had lower ED charges ($2,200 vs $1,520, *P* < 0.001). Visits to PEDs had lower adjusted odds of critical procedures (adjusted odds ratio [aOR] 0.74, 95% confidence interval [CI] 0.62–0.87), transfers (aOR 0.14, 95% CI 0.04–0.56), and mortality (aOR 0.38, 95% CI 0.19–0.75) compared to GEDs.

**Conclusion:**

Care for children with neuro CCCs in a pediatric ED is associated with less resource utilization and lower rates of transfer and mortality. Identifying features of PED care for neuro CCCs could lead to lower costs and mortality for this population.

Population Health Research Capsule What do we already know about this issue?

*Children with chronic neurologic diseases are at risk for severe illness and poor outcomes in the emergency department.*

What was the research question?

*Does emergency care differ between general (GED) and pediatric EDs (PED) for children with chronic neurologic disease?*

What was the major finding of the study?

*Chronic neurological pediatric visits in PEDs had lower odds of mortality in the ED (aOR 0.4, 95% CI 0.2–0.8) compared to GEDs (0.04% vs 0.13%).*

How does this improve population health?

*Identifying features of pediatric ED care for children with chronic neurologic conditions could improve mortality in this high-risk population.*

## INTRODUCTION

Approximately 30 million emergency department (ED) visits are made by children in the United States annually.^1^ Previous studies have shown that most pediatric patients receive emergency care in general EDs (GED), where less pediatric experience and lower pediatric emergency readiness may compromise care.^2^ ^,^ ^3^ Care in GEDs may be less likely to follow evidence-based guidelines for common pediatric conditions such as head trauma, croup, and asthma. In addition, GEDs may have higher rates of potentially avoidable transfers (given the likelihood of transfer when the patient did not present initially to a specialty hospital) and higher mortality in critical illness.^4^ ^–^ ^6^

Children and young adults with chronic, severe neurologic conditions are a medically vulnerable population with poor functional status and high rates of comorbid disease and need for technology assistance (ie, gastrostomy tube or ventricular peritoneal shunts). These characteristics make this population at risk of frequent ED utilization, unique medical presentations, and high acuity of illness.^7^ ^–^ ^11^ Prior studies have found children with neurologic disease account for up to 13% of inpatient pediatric admissions, and one third of inpatient pediatric healthcare costs.^7^ ^,^ ^8^ Although GEDs provide most emergency care for children, emergency medicine (EM) trainees and GED clinicians have less experience and confidence in providing care to pediatric patients compared to treating adults.^12^ ^,^ ^13^ Despite growing evidence that children with chronic neurologic conditions are at risk of frequent ED usage, there is limited data comparing the distribution, characteristics, and outcomes of emergency care for this specific population between GEDs and pediatric EDs (PED).

In this study we aimed to describe the national estimates of children and young adult patients with neurologic complex chronic conditions (neuro CCC) and compare the characteristics and outcomes of ED visits in GEDs and PEDs. We hypothesized that PED visits would be associated with lower rates of resource utilization, transfer, and mortality.

## METHODS

### Study Design and Data Source

We conducted a cross-sectional analysis to identify the frequency and characteristics of ED visits among patients aged 0–21 years with neuro CCCs between 2011–2014 in the Nationwide Emergency Department Sample (NEDS). The NEDS is part of the Healthcare Cost and Utilization Project database that is sponsored by the Agency for Healthcare Research and Quality. It is the largest all-payer, nationally representative ED database in the United States and contains a 20% stratified sample of hospital-based EDs. The sampling strategy deliberately encompasses between 945–955 hospital-based EDs in 24–34 states and approximately 120–137 million weighted visits to the ED annually.^14^ Hospital-based EDs are stratified by US census region, trauma designation, urban-rural location, hospital ownership, and teaching status. Additionally, as no patient identifiers are available, individual patients cannot be tracked longitudinally, and encounters that originated in one facility and were transferred will have a separate encounter in the receiving facility. The unit of analysis is the ED visit. The study was submitted to the Seattle Children’s Institutional Review Board (IRB) and was determined to be IRB exempt.

We included all ED visits made by patients 0–21 years of age with an International Classification of Diseases, 9^th^ Rev (ICD-9) diagnosis consistent with a neuro CCC.^15^ The classification of CCCs, originally developed by Feudnter and colleagues, is an organ-system based classification of complex diseases of childhood.^15^ We excluded ED visits that were missing age, primary diagnosis. or the primary diagnosis was invalid.

Our primary predictor variable was the category of ED, either GED or PED. The NEDS does not specifically designate PEDs; thus, consistent with similar published reports, we designated PEDs as those where ≥75% of visits were children <18 years of age. All other EDs were categorized as GEDs.^3^ ^,^ ^5^ ^,^ ^16^ ^–^ ^18^ Of note, while we defined PED based upon proportion of visits made by children <18 years, we included encounters in this study up to age 21, as patients with chronic medical conditions often continue to seek care in PEDs into young adulthood as they transition to adult care.^2^ ^,^ ^3^ Patient-level predictors included the following: 1) demographics, insurance payer, median income quartile, and the urban-rural classification of the patient’s area of residence; 2) neuro CCC diagnostic category; 3) number of non-neuro CCCs); 4) presence of technology assistance and CCC ICD-9 codes.^15^ We identified specific technologies, including ventricular shunts, feeding tubes (gastrostomy, gastro-jejunostomy, and jejunostomy tubes) and tracheostomies, specifically using the corresponding technology assistance codes.^15^ Hospital-level predictors included teaching status of the ED, trauma center designation, and hospital region.

Our primary outcomes of interest were resource utilization, severity of illness presentation, disposition (admission and transfer), and mortality. Resource utilization was assessed through ED charges, frequency of procedures performed, and diagnostic imaging. As the NEDS provides only facility charges, and cost-to-charge ratios were not available for the years selected, we report total charges for the ED and inpatient stay. This approach is consistent with prior published studies.^19^ ^,^ ^20^ We used total number of current procedural terminology (CPT) codes, rather than ICD-9 procedural codes, to assess procedure frequency as a significantly higher proportion of ED visits had CPT codes available. Diagnostic imaging (including radiograph, ultrasound, computed tomography (CT) or magnetic resonance imaging (MRI) and cross-sectional imaging (CT or MRI only) was reported based on the CPT and ICD-9 procedural codes associated with the ED visit.

We assessed the outcome of severity of clinical presentation using severity classification scores (SCS) and critical procedure performance.^2^ ^,^ ^21^ The SCS is a Pediatric Emergency Care Applied Research Network consensus-derived diagnostic system that relies on the most severe ICD-9 diagnostic codes attached to each record to assign each ED visit a severity score. The severity score ranges from 1 (minimal resources used) to 5 (maximal resources used).^21^ Critical procedures were defined as the presence of an ICD-9 code for endotracheal intubation, central line placement, and chest tube placement as previously described in the literature.^22^ ^,^ ^23^ Mortality was categorized as (1) ED mortality and (2) visit mortality (death at any point during ED visit or hospitalization).

### Statistical Analysis

We incorporated sampling weights to consider the significant survey design and sampling procedures of the NEDS. Descriptive statistics, including frequencies, proportions and sums as appropriate, were used to summarize patient and hospital characteristics. We made comparisons using chi-square or ANOVA test for categorical variables, and *t*-tests for continuous variables. Multivariable logistic regression was performed for five different ED outcome variables (admission, transfer, transfer or admission, mortality, and critical procedure performance). Predictor variables included in logistic regression were patient-level variables (demographics, number of CCCs, technology assistance, SCS score), and hospital-level predictors (trauma center designation, geographic location, PED vs GED). Results were reported as adjusted odds ratios (aOR) and 95% confidence intervals (CI).

## RESULTS

Of the estimated 141 million weighted ED visits made by patients aged 0–21 years in the 2011-2014 NEDS, 387,987(0.3%) had a neuro CCC diagnosis. Most neuro CCC ED visits occurred in GEDs (74.9%), and the remainder occurred in PEDs (25.1%). Neuro CCCs visits represented proportionately more of all 0–21-year-old PED visits compared to GED visits (1.0% vs 0.2%). The patient-level characteristics of neuro CCC ED visits are shown in Table 1. Younger patients (ages ≤9 years) represented proportionately more of PED than GED visits (63 vs 48%, *P* < 0.01). There was a predominance of males in both GED and PED visits (55.8% vs 55.9%). The primary payer for most GED and PED visits was Medicaid (56.1% vs 60.6%), and income quartile (not shown) was not significantly different between GEDs and PEDs.

**Table 1.** Selected patient, hospital, and visit characteristics involving visits to general and pediatric emergency departments for neuromuscular complex chronic conditions.

N (%)	General ED (n = 290,641)	Pediatric ED (n = 97,346)	All ED visits (n = 387,987)	P value
Patient characteristics				
Age in years				
0–9	139,249 (47.9%)	61535 (63.2%)	200,706 (51.7%)	<0.001
10–17	79,886 (27.5%)	29,138 (29.9%)	109,024 (28.1%)	
18–21	71,505 (24.6%)	6,712 (6.9%)	78,257 (20.2%)	
Urbanicity				
Large metro	142,792 (49.1%)	72,560 (74.5%)	215,353 (55.5%)	0.18
Medium/small metro	100,482 (34.5%)	15,978 (16.4%)	116,460 (30.0%)	
Non-metro/unknown	47299 (16.3%)	8808 (9.0%)	56107 (14.5%)	
Primary payer	
Medicaid	162,965 (56.1%)	58,950 (60.6%)	221,916 (57.2%)	0.50
Private insurance	98780 (34.0%)	30,832 (31.7%)	129,612 (33.4%)	
Medicare/other	28,807 (9.9%)	7393 (7.6%)	11,868 (9.4%)	
Complexity				
>1 additional CCC	69,038 (21.0%)	32,037 (32.9%)	93,044 (24.0%)	<0.001
Technology assistance	143,788 (49.5%)	63,707 (65.45%)	207,495 (53.5%)	<0.001
Hospital characteristics				
Teaching hospital	195,506 (67.3%)	97,035 (99.7%)	292,542 (75.4%)	<0.001
Trauma center (I/II)	165,244 (56.9%)	90,905 (93.4%)	256,149 (66.2%)	<0.001
Large metro location	150,888 (51.9%)	80,818 (83.0%)	231,744 (59.7%)	0.015
Visit characteristics				
Disposition				
Admission	124,350 (42.8%)	53,659 (55.1%)	178,008 (45.9%)	<0.001
Transfer	27,392 (9.4%)	1,242 (1.3%)	28,633 (7.4%)	<0.001
Death in the ED	380 (0.13%)	47 (0.04%)	427 (0.11%)	0.003
Critical procedures^1^				
Endotracheal tube	20,059 (8.3%)	4,220 (5.7%)	24,278 (7.7%)	0.003
Central venous line	11,210 (4.6%)	3,873 (5.3%)	15,115 (4.8%) 1,763 (0.6%)	0.32
Chest tube	1,511 (0.6%)	255 (0.3%)	77,574 (20.0%)	0.004
Severity classification score				
<3	60,545 (20.8%)	17,030 (17.5%)	192,480 (49.6%)	0.007
4	138,084 (47.5%)	54,357 (55.8%)	116,823 (30.1%)	<0.001
5	91,060 (31.3%)	25,762 (26.4%)	$2,031 (1170–3743)	0.002
ED charges, median (IQR)	$2,200 (1237–3943)	$1,520 (873–2783)	$2,031 (1170–3743)	<0.001

1 Critical procedures included both current procedural terminology (CPT) and International Classification of Diseases, 9th Rev (ICD-9) procedure codes. Visits with CPT/ICD-9 procedure codes listed, Total n = 367,108; general ED n = 241,401; pediatric ED n = 73,947. *ED*, emergency department; *CCC*, complex chronic conditions; *IQR*, interquartile range.

There was a high rate of comorbid chronic conditions overall in children with neuro CCCs, with one in four ED visits associated with at least one non-neurologic CCC (93,075, 24%, Table 1). The medical complexity of neuro CCC visits was higher in PEDs compared to GEDs, with 32.9% of patients in PEDs with at least one additional CCC compared to 21% in GEDs. Technology assistance was more frequent in PED than GED encounters (65.5% vs 49.5%) and was comprised mostly of ventricular shunts (161,868, 41.7%), feeding tubes (56,568, 14.6%) and, less commonly, tracheostomies (17,653, 4.6%). Supplemental Table 1 demonstrates the frequency of subcategories of neuro CCCs and the most common categories of non-neuro CCCs.

Hospital characteristics are also shown in Table 1. Over 80% of PED visits were in metropolitan locations, teaching facilities, and Level I/II trauma centers, consistent with underlying differences between these two categories of EDs.^2^ ^,^ ^18^ Regionally, PED visits were predominantly from the West, while GED visits were predominantly from the South. The Northeast region accounted for the lowest proportion of visits, 18.9% of neuro CCC ED visits overall and only 1.4% of PED visits (data not shown).

Characteristics of emergency visit care for neuro CCC ED visits are demonstrated in Table 1. Severe illness presentations were common; 30.1% of visits had a SCS 5 indicating critical illness. The PEDs had fewer SCS 5 presentations than GEDs, (26.4% vs 31.3% vs. *P* = 0.002), and more SCS 4 presentations than GEDs (55.8% vs. 47.5%, *P* = < 0.001). PEDs visits had fewer overall procedures performed (0 procedures performed in 34.9% PED vs13.4% in GEDs (*P* = 0.048) and less imaging (45.7% vs 24.4%, not significant). Endotracheal intubation was the most frequently performed critical procedure and occurred less frequently in PEDs compared to GEDs (5.7% vs 8.3%, *P* = 0.003).

Median ED charges were significantly lower in PEDs compared to GEDs (*P* < 0.001). Visits to PEDS, had higher proportion of admissions (55.1% vs 42.8%, *P* < 0.001) and lower proportion of transfers (1.3% vs 9.4%, *P* < 0.001). In the combined outcome of admission or transfer, there were no significant differences between PEDs and GEDs (52.2% vs 56.4%, *P* = 0.09). Death in the ED was an infrequent outcome, representing only 0.11% of visits. However, ED mortality was lower for PED visits compared to GED visits (0.02% vs 0.11%, *P* = 0.003). Visit mortality (death at any point during ED or inpatient stay) was similarly lower for PED visits compared to GEDs (1.27% vs 2.39%, *P* = 0.003).

Table 2 summarizes results of our logistic regression models to explore the relationship between the category of ED and visit disposition and critical procedure performance. The PED visits had significantly lower adjusted odds of transfer compared to general EDs (aOR 0.14, 95% CI 0.04–0.56). Conversely, PEDs had a significantly higher adjusted odds of admission (aOR 1.52, 95% CI 1.19–1.96). Additional predictors in admission and transfer models included presence of non-neurologic CCCs, increased severity of illness, rural/fringe metropolitan residences, and in those whose insurance was self-pay. For the combined outcome of admission or transfer, there was no significant difference between PEDs and GEDs (aOR 1.07, 95% CI 0.86–1.33).

**Table 2.** Logistic models for outcome of admission, transfer, and the combined outcome of admission or transfer for emergency department visits for neurologic complex chronic conditions.

	Admission adjusted OR (95% CI)	Transfer adjusted OR (95% CI)	Admission or transfer adjusted OR (95% CI)
Age	1.01 (1.01–1.02)	0.94 (0.93–0.95)	0.99 (0.98–1.00)
Female gender	0.93 (0.89–0.97)	1.06 (1.00–1.13)	0.95 (0.92–0.99)
Insurance payer			
Private insurance	Referent	Referent	Referent
Medicaid	0.85 (0.78–0.93)	1.10 (0.99–1.23)	0.88 (0.82–0.95)
Medicare	0.88 (0.71–1.10)	0.84 (0.57–1.24)	0.86 (0.69–1.07)
Urbanicity			
Central metro	Referent	Referent	Referent
Small metro	0.74 (0.58–0.96)	2.33 (1.75–3.09)	0.96 (0.78–1.18)
Non-metro	0.79 (0.54–0.89)	2.98 (2.28–3.9)	1.01 (0.84- 1.21)
Additional non-neurologic CCCs	2.56 (2.31–2.86)	0.43 (0.38–0.49)	2.25 (2.05–2.47)
Feeding tube	1.30 (1.17–1.45)	0.82 (0.69–0.98)	1.23 (1.1–1.37)
Ventricular shunt	1.01 (0.89–1.14)	0.54 (0.46–0.63)	0.84 (0.76–0.93)
Tracheostomy	0.59 (0.48–0.71)	2.8 (2.11–3.70)	0.71 (0.6–0.85)
Severity classification score	2.52 (2.40–2.64)	1.9 (1.75–2.06)	3.0 (2.86–3.31)
Pediatric ED	1.52 (1.19–1.96)	0.14 (0.04–0.56)	1.07 (0.86–1.33)

*OR*, odds ratio; *CI*, confidence interval; *CCCs*, complex chronic conditions; *ED*, emergency department.

In the adjusted models of critical procedures (Table 3), PED visits had lower odds of critical procedures (aOR 0.74, CI 0.62–0.87) compared to GED visits. Overall, increased severity of illness was associated with a dramatically increased odds of critical procedures (aOR11.9, 95% CI 10.3–13.6). Non-neurologic CCCs were also associated with increased of critical procedures performance (aOR 1.51, 95% CI 1.42–1.59). The ED visits in which a patient had a tracheostomy had lower odds (aOR 0.48, 95% CI 0.38–0.59) of critical procedure as compared to those without a tracheostomy; other forms of technology assistance were not significantly different.

**Table 3.** Logistic models for the outcomes of critical procedure performance, death in the emergency department or death at any point in the visit (visit mortality).

	Critical procedure performance adjusted OR (95% CI)	ED mortality adjusted OR (95% CI)	Visit mortality adjusted OR (95% CI)
Age	1.0 (0.994–1.005)	0.97 (0.94–1.0)	1.03 (1.02 –1.04)
Female gender	0.81 (0.76–0.85)	0.63 (0.39–1.0)	0.76 (0.69–0.84)
Insurance payer			
Private insurance	Referent	Referent	Referent
Medicaid	1.03 (0.96–1.11)	1.04 (0.65–1.67)	0.66 (0.58–0.75)
Medicare	0.85 (0.62–1.15)	1.09 (0.15–8.24)	0.19 (0.09–0.36)
Urbanicity			
Central metro	Referent	Referent	Referent
Small metro	0.81 (0.70–0.94)	1.12 (0.54–2.31)	0.94 (0.74–1.12)
Non-metro	0.65 (0.56–0.75)	2.0 (0.89–4.54)	0.90 (0.72–1.12)
Non-neurologic CCCs	1.51 (1.42–1.59)	0.86 (0.55–1.33)	2.73 (2.54–2.94)
Feeding tube	0.92 (0.83–1.02)	1.34 (0.49–3.67)	0.23 (0.18–0.30)
Ventricular shunt	0.91 (0.81–1.02)	0.34 (0.18–0.64)	0.33 (0.28–0.40)
Tracheostomy	0.48 (0.38–0.59)	2.65 (0.77–9.17)	0.22 (0.15–0.31)
Severity classification score	11.81 (10.28–13.56)	N/A	N/A
Pediatric ED	0.74 (0.62–0.87)	0.38 (0.19–0.75)	0.62 (0.46–0.83)

*OR*, odds ratio; *CI*, confidence interval; *CCCs*, complex chronic conditions; *ED*, emergency department.

The logistic models for ED mortality and visit mortality are also shown in Table 3. Severity of illness scores were not included in the adjusted models of mortality, as there was collinearity with this variable and the outcome. The adjusted odds of ED mortality was significantly lower for PED visits (aOR 0.37, CI 0.19–0.73) compared to GED visits. Patients with ventriculorperitoneal shunts had a lower adjusted odds of mortality compared to those without. Similarly, in the model of overall visit mortality, PED visits had a lower odds of visit mortality compared to GEDs (aOR 0.62, *P* < 0.001). The presence of non-neurologic CCCs was predictive of increased odds of mortality, while all forms of technology assistance had lower adjusted odds of visit mortality.

## DISCUSSION

In this national sample of ED visits, we estimate 387,000 annual ED visits were made by patients aged 0–21 years with neurologic complexity between 2011–2014. Neuro CCC patients had high rates of medical complexity and technology dependence, and often presented with severe illness to the ED. Most of the emergency care for this population occurred in GEDs, where visits had higher rates of diagnostic testing, critical procedures, and ED-associated charges. After adjustment for differences in demographics, comorbidities, and severity of illness presentation, GEDs had higher rates of transfer. However, there were no significant differences between GEDs and PEDs in a combined model of admission or transfer. Adjusted odds of critical procedure performance, ED mortality, and overall visit mortality were higher in GEDs compared to PEDs.

Prior research has demonstrated that children with complex chronic illnesses have a high risk of critical illness and poor outcomes during emergencies.^17^ ^,^ ^18^ ^,^ ^24^ ^,^ ^25^ Our study adds to the literature by demonstrating that among a national sample of neuro CCC ED visits, an estimated 30% presented with critical severity of illness (SCS 5) and 7.7% required endotracheal intubation. In contrast, in a national sample of all-comer PED visits in the NEDS in which only 5% of patients had ≥1 complex chronic condition, only 0.5% of visits had a critical severity score of 5 and only 0.15% required intubation.^2^ The comparatively much higher severity measures found in our study population further supports the high-acuity ED needs among children with neurologic complexity as compared to a general pediatric population.

Our work demonstrates that most emergency care for children with neuro CCCs occurs in GEDs rather than specialized pediatric centers, congruent with prior characterizations of emergency care for children with CCCs.^2^ Prior research using nationally representative datasets has shown that PEDs may perform better across several quality-of-care metrics, including less diagnostic testing in asthma, fewer antibiotics for viral infections, and lower mortality in critical illnesses such as cardiac arrest and sepsis.^4^ ^,^ ^5^ ^,^ ^25^ Our study expands upon this existing literature, by characterizing the disparities in characteristics of emergency care in GEDs for children with chronic neurologic diseases. These findings suggest there may be some specific benefits to PED care for certain high-risk, medically fragile populations, such as those with neurologic complexity.

Some of the differences we observed between GEDs and PEDs may be due to unmeasured influences of a pre-transfer evaluation and stabilization. As this dataset has no patient identifiers and does not allow for longitudinal assessment of patient care, we were unable to identify which ED visits were self-referred vs transferred from another ED. However, among the 28,633 transferred encounters in this study, 27,392 (95.7%) originated in a GED, and it is likely many of these encounters were transferred to a PED. Once these patients reached the receiving facility, they likely had reduced requirements for additional diagnostic testing or critical interventions, which could account for the comparatively lower ED costs and procedure frequency we observed in PED encounters. Additionally, other factors related to the transfer process may have influenced procedure rates in GEDs. For instance, referring emergency physicians might have chosen to intubate patients with a higher risk of respiratory decompensation before the transfer, potentially contributing to the relatively higher intubation frequency seen in GEDs. To gain a deeper understanding of the origins of the observed variations in ED costs and outcomes between GEDs and PEDs, future studies incorporating longitudinal patient data are needed.

It is worth noting that ED visits made by children with neuro CCCs had high rates of technology assistance overall, and in our logistic models patients with tracheostomy and ventricular shunts had lower odds of visit mortality. Although technology assistance in large population studies of all-comer pediatric patients has been identified as a risk factor for severe illness and mortality, in children with neurologic diseases there is evidence that technology assistance may be protective.^26^ ^,^ ^27^ In a 2019 Canadian study of children with medical complexity, technology assistance was associated with lower odds of visit mortality in children with neurologic impairment and those with multiple CCCs.^8^ Similarly, a 2015 analysis from Hong Kong found that in children with severe neurologic diseases, tracheostomy was associated with lower odds of mortality.^28^ Additionally, inherent differences in the type of neurologic complexity between patients with and without these forms of technology assistance may help explain the observed differences in mortality.

We hypothesize the higher rates of transfer and lower rates of admission in GEDs are likely secondary to limited inpatient pediatric capabilities at these centers, thus necessitating transfer.^6^ ^,^ ^7^ ^,^ ^29^ This hypothesis is supported by our finding that the combined outcome of admission and transfer in our logistic models showed no differences between GEDs and PEDs. There is increasing evidence that pediatric inpatient care is increasingly limited in community hospitals, resulting in increased regionalization of hospital pediatric care.^30^ This is likely to be particularly true for children with neurologic complexity, who may require specialist consultation only available in pediatric centers.

These findings have important implications for the delivery of pediatric emergency care to medically vulnerable patients in the United States. Despite the increasing regionalization of inpatient pediatric care, emergency care for children is likely to continue to occur predominantly in GEDs given the geographic limitations in access to specialized pediatric emergency centers for many patients. Thus, ensuring adequate education and preparation for emergency conditions in complex pediatric patients in community and rural EDs is critical. Experience caring for critically ill, medically complex pediatric patients is lacking for many EM trainees and represents a target for ongoing educational efforts.^12^ ^,^ ^13^

Simulation interventions, such as those delivered by the IMPACTS network. are another possible intervention to help improve the care of this complex population by non-pediatric clinicians in community ED settings.^31^ Pediatric emergency telemedicine may be another potential strategy to improve the quality of care received by complex pediatric patients in GEDs. Improvements in this technology, wider availability of telemedicine clinicians, and increasing acceptance of this format of care may ultimately address disparities in access to care by making specialized pediatric emergency physicians more available.^32^ ^–^ ^34^

## LIMITATIONS

This study has several important limitations. First, we used data from 2011–2014, which may impact how translatable these findings are to the present. Increasing regionalization of care in the last 10 years may have impacted overall distribution of pediatric neuro CCC ED care between GEDs and PEDs and potentially an increased frequency of transfers. Increased efforts toward pediatric readiness in GEDs during this time frame could also have improved critical illness outcomes in some GEDs. Additionally, this work relies on large amounts of administrative data, which is susceptible to errors in data processing and variability in coding. We used ICD–9 codes to identify the population of neuro CCC visits, and the ICD–9 codes ascribed to an encounter only pertain to the currently recorded ED diagnoses and may not represent all pre-existing conditions. Thus, this work likely underestimates the true frequency of neuro CCC ED visits, particularly for lower acuity treat- and-release visits.^2^

Additionally, using the proportion of pediatric patients seen within an ED to determine PED designation has its own limitations. Specifically, if a PED and GED are financially linked (common in academic institutions that share the same campus) the visits from these two institutions will often be grouped as a single hospital in the NEDS. This results in some PEDs being grouped together with GEDs, using our categorization system. Given the collinearity of the outcome of mortality with SCS, we did not include this in our modeling, and thus differences in mortality between GEDs and PEDs may in part be due to unmeasured differences in severity of illness. Lastly, mortality was overall an infrequent outcome, and thus any broad interpretations of this finding should be taken with caution.

## CONCLUSION

This study is the first in our knowledge to describe the national state of emergency care for children with neurologic complex chronic conditions in both pediatric and general EDs. Our findings demonstrate that most emergency care for children with neuro CCCs occurs in GEDs, and that GEDs had higher rates of procedures and charges, transfers, and mortality as compared to PEDs. As these patients are likely to continue to predominantly receive emergency care in GED settings, interventions to ensure appropriate training and preparation of general emergency physicians for children with neurologic complexity is needed. Additionally, further research efforts to explore the impact of pediatric emergency telemedicine support on improved quality of care for medically complex patients is needed.

## Supplementary Information




