# Supplementary material for: National Characteristics of Emergency Care for Children with Neurologic Complex Chronic Conditions
Source: West J Emerg Med. 2024 Feb 9;25(2):237–45. doi: 10.5811/westjem.17834 (PMC11000559; doi:10.5811/westjem.17834)
Supplement: Supplementary file 1 [file wjem-25-237-s001.docx]

**Supplemental Table 1.** Neurologic complex chronic condition (CCC) subcategories and categories of non-neuro CCCs (%). Subcategories were not mutually exclusive; so visits may have had one or more neuro CCC subcategories.

|  | General ED | Pediatric ED | All EDs |
| --- | --- | --- | --- |
| Neurologic CCC categories, n (%)  Neurologic devices*  CNS degeneration*  Epilepsy  Other CNS diseases  Brain/spinal malformations*  Cerebral palsy*  Intellectual disability^±^  Muscular dystrophy*  Movement disorder*  Cerebral artery occlusion* | 108,636 (37.3%)  78,955 (27.1%)  58,314 (20.1%)  46,248 (15.9%)  35,152 (12.1%)  36,432 (12.5%)  19,593 (6.7%)  15,248 (5.3%)  10,747 (3.7%)  5,859 (2.0%) | 47,257 (48.5%)  31,388(32.2%)  18,740 (19.2%)  15,248 (15.7%)  19,515 (20.0%)  17,459 (17.9%)  3,880 (4.0%)  3,763 (3.9%)  2,677 (2.8%)  1,164 (1.2%) | 155,932 (40.2%)  110,343 (28.4%)  77,054 (19.9%)  61,535 (15.9%)  54,667 (14.1%)  53,891 (13.9%)  23,473 (6.1%)  19,011 (4.9%)  13,424 (3.5%)  7,023 (1.2%) |
| Category of non-neuro CCC, n (%)  Gastrointestinal*  Respiratory  Metabolic*  Renal*  Cardiovascular  Hematologic  Congenital*  Neonatal*  Transplant  Malignancy | 33,949 (11.7%)  13,618 (4.7%)  8,807 (3.0%)  8,187 (2.8%)  7,100 (2.4%)  3,414 (1.2%)  3,220 (1.1%)  2,600 (0.9%)  1,125 (0.4%)  854 (0.3%) | 19,399 (19.9%)  6,014 (6.2%)  4,927 (5.1%)  3,879 (4.0%)  2,755 (2.8%)  2,367 (2.4%)  2250 (2.3%)  1,163 (1.2%)  582 (0.6%)  543 (0.6%) | 53,358 (13.8%)  19,632 (5.1%)  13,114 (3.4%)  12,687 (3.3%)  9,855 (2.5%)  5,587 (1.4%)  4,850 (1.3%)  4,578 (1.2%)  1,707 (0.4%)  1,436 (0.4%) |

**P*<0.01.

^±^Original neuromuscular CCC classification was mental retardation.

*ED*, emergency department; *CCC*, complex chronic condition; *CNS*, central nervous system.
